# The Efficacy and Safety of Nab‐Paclitaxel Plus Anlotinib in Small‐Cell Lung Cancer for Second‐Line Therapy

**DOI:** 10.1111/1759-7714.70290

**Published:** 2026-05-11

**Authors:** Li‐Jun Tian, Fei‐Fei Zhou, Min Dai, Jun‐Li Liu, Qi‐Sen Guo

**Affiliations:** ^1^ Department of Oncology Shandong University Cancer Center Jinan Shandong China; ^2^ Department of Oncology Binzhou Medical University Hospital Binzhou Shandong China

## Abstract

**Background:**

Extensive Small‐cell lung cancer (ES‐SCLC) has a poor prognosis following the failure of first‐line therapy based immune checkpoint inhibitors. This study aimed to evaluate the efficacy and safety of nab‐paclitaxel combined with anlotinib as second‐line treatment for relapsed SCLC.

**Methods:**

Patients were divided into two groups: patients receiving 125 mg/m^2^ of nab‐paclitaxel on Days 1 and 8, repeated every 3 weeks for six cycles (the NAP group) and patients receiving 125 mg/m^2^ of nab‐paclitaxel on Days 1 and 8 accompanied with 12 mg/day of anlotinib for 14 days, repeated every 3 weeks for up to six cycles, followed by maintenance anlotinib until disease progression or unacceptable toxicity (the ANNAB group). The primary endpoints were progression‐free survival (PFS) and overall response rate (ORR). The secondary endpoints were overall survival (OS) and safety.

**Results:**

Between January 1, 2023, and July 31, 2024, 48 patients were enrolled into the study. The median PFS was 6.0 months in the ANNAB group and 4.7 months in the NAP group (*p* = 0.0004). ORR was significantly higher in the ANNAB group than in the NAP group (37.5% vs. 8.3%, *p* = 0.0363). The median OS was 10.0 months in the ANNAB group compared to 7.3 months in the NAP group (*p* < 0.0001). There were no significant differences in adverse events between the two groups.

**Conclusions:**

The combination of nab‐paclitaxel and anlotinib as second‐line treatment for recurrent SCLC demonstrated promising efficacy and an acceptable toxicity profile, suggesting its potential as a viable therapeutic strategy.

## Graphical abstract

Nab‐paclitaxel plus anlotinib significantly improved PFS, ORR, and OS with manageable safety in relapsed SCLC after first‐line chemoimmunotherapy, offering a promising second‐line therapeutic option.

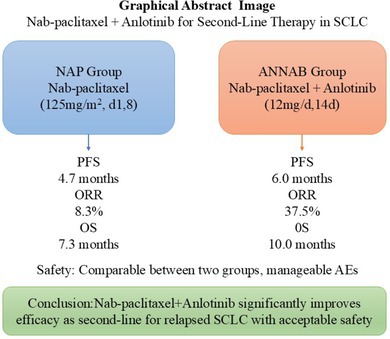

**Abbreviations**
AEs: adverse events
CR: complete response
DCR: disease control rate
ECOG: Eastern Cooperative Oncology Group Performance Status
ES‐SCLC: extensive small‐cell lung cancer
ICIs: immune checkpoint inhibitors
NCCN: National Comprehensive Cancer Network
NCI‐CTCAE: National Cancer Institute's Common Terminology Criteria for Adverse Events
NMPA: National Medical Products Administration
ORR: objective response rate
ORR: overall response rate
OS: overall survival
PD: progressive disease
PFS: progression‐free survival
PR: partial response
RECIST: response evaluation criteria in solid tumors
SCLC: small‐cell lung cancer
ULN: upper limit of normal
WBC: white blood count

## 1 Introduction

Small‐cell lung cancer (SCLC) accounts for approximately 13%–15% of newly diagnosed lung cancer cases worldwide. It is considered an aggressive disease with a poor prognosis [1]. Approximately 70% of patients present with extensive‐stage SCLC (ES‐SCLC) at initial diagnosis, which is associated with significantly worse survival outcomes than limited‐stage disease [1]. The standard first‐line therapy for ES‐SCLC, consisting of platinum‐based chemotherapy combined with etoposide, has remained unchanged for the past three decades and has resulted in a median overall survival (OS) of approximately 10 months [2]. Although the introduction of immune checkpoint inhibitors (ICIs), such as atezolizumab and durvalumab, into the first‐line treatment regimen for ES‐SCLC has led to a two‐month improvement in median OS [3, 4], the overall prognosis remains unsatisfactory.

There is no standardized treatment after the first‐line chemoimmunotherapy. There is significant heterogeneity in second‐line treatment recommendations across different countries based on the absence of ICIs in the first‐line therapy. For example, topotecan is commonly used in Europe and North America [5], while amrubicin is preferred in Japan [6]. Despite variations in approved second‐line therapy regimens, low response rates result in short relief periods. Moreover, severe hematologic toxicity and poor drug accessibility in China have limited the use of these drugs. Despite the clinical application of paclitaxel‐based chemotherapy, it is often designated as a category 2A recommendation in current evidence‐based clinical guidelines. The nanoparticle albumin‐bound (nab)‐paclitaxel (nab‐paclitaxel), a new solvent‐free formulation of paclitaxel, has been demonstrated to be more effective than paclitaxel in treating relapsed SCLC in some retrospective studies [7, 8, 9]. Although a prospective Phase 2 study demonstrated the efficacy of nab‐paclitaxel monotherapy as a second‐line therapy for relapsed SCLC, the overall outcome remained unsatisfactory [10].

Anlotinib, a novel anti‐angiogenic tyrosine kinase inhibitor independently developed in China, was approved as a third‐line treatment by the National Medical Products Administration (NMPA) in 2019 based on the ALTER 1202 study, demonstrating improved progression‐free survival (PFS) and OS [11]. Anlotinib also showed potent tumor growth inhibition as second‐line therapy for relapsed SCLC, especially in terms of therapeutic integration. Han et al. demonstrated that anlotinib combined with ICIs as a second‐line treatment regimen for relapsed SCLC shows promising antitumor activity [12]. The available clinical evidence remains insufficient to support the continued use of ICIs following disease progression in ES‐SCLC, and no demonstrable survival benefit was observed in the current study.

This therapeutic dilemma is particularly relevant given the lack of established second‐line treatment options following first‐line chemoimmunotherapy regimens, highlighting the urgent need for standardized therapeutic algorithms in this clinical scenario. In this retrospective study, we aimed to investigate the efficacy and safety of the combined‐modality treatment of anlotinib and nab‐paclitaxel (defined as an ANNAB group) compared with nab‐paclitaxel monotherapy (defined as an NAP group) as a second‐line treatment for patients diagnosed with relapsed SCLC.

## 2 Methods

### 2.1 Patients

From January 1, 2023, to July 31, 2024, Binzhou Medical University Hospital enrolled patients aged 18–75 years with a pathologically confirmed diagnosis of ES‐SCLC. The key inclusion criterion was histologically or cytologically confirmed SCLC with disease progression after first‐line chemoimmunotherapy treatment with platinum‐based therapy. Additionally, they needed to have an Eastern Cooperative Oncology Group Performance Status (ECOG) PS of 0–1 at enrollment [13] and measurable lesions based on the Response Evaluation Criteria in Solid Tumors (RECIST) version 1.1. Furthermore, eligible patients were required to have a well‐functioning bone marrow (hemoglobin ≥ 9.0 g/L, absolute neutrophil count ≥ 1.5 × 10^9^/ × L, platelet count ≥ 90 × 10^9^/ × L, white blood count (WBC) ≥ 3.0 × 10^9^/ × L), liver function (total bilirubin ≤ 2.0 mg/dL, albumin ≥ 35 g/L); aspartate and alanine transaminase, ≤ 2 times the upper limit of normal (ULN), ≤ 3 times ULN with liver metastases, and kidney function (serum creatinine ≤ 1.5 mg/dL). Asymptomatic or controlled brain metastases were also observed. Patients who had previously received anti‐angiogenic agents, uncontrolled hypertension (blood pressure ≥ 160/100 mmHg), risk of bleeding due to tumors near major blood vessels, uncontrolled serious medical conditions (e.g., unstable angina), or intolerance to nab‐paclitaxel or anlotinib were excluded.

This study adhered to the ethical guidelines of the Ethics Committee of the Binzhou Medical University Hospital. All the procedures involving human subjects were conducted in accordance with the Declaration of Helsinki and its amendments. Written informed consent was obtained from all participants prior to their enrollment in the study. Primary chemotherapy resistance was defined as disease progression during or within 180 days of the first‐line platinum‐based treatment. Conversely, primary chemotherapy sensitivity was defined as disease progression at least 180 days after the completion of first‐line treatment.

### 2.2 Treatment Allocation

This was a retrospective, nonrandomized study. Patients were assigned to receive either ANNAB or NAP based on the physician's clinical assessment, patient comorbidities, prior treatment‐related toxicities, and patient preference. Treatment allocation was not randomized, which may lead to potential selection bias.

### 2.3 Treatment Schedule

Following the failure of first‐line chemoimmunotherapy, treatment allocation to either the NAP or ANNAB group was conducted. The treatment regimen consisted of intravenous nab‐paclitaxel (125 mg/m^2^ within 30 min on Days 1 and 8) administered in combination with 12 mg of anlotinib, adhering to the 21‐day cycle structure recommended by the National Comprehensive Cancer Network (NCCN) guidelines in the ANNAB group. Following the six cycles of the ANNAB regimen, patients who demonstrated a positive response on radiological assessment were eligible to continue receiving anlotinib monotherapy until disease progression or treatment intolerance. In the NAP group, the patients received intravenous nab‐paclitaxel (125 mg/m^2^ within 30 min on Days 1 and 8) for up to six cycles. Dose modifications for anlotinib and nab‐paclitaxel were implemented by the attending physician when necessary, following the protocol‐defined criteria established for the study drugs. The National Cancer Institute's Common Terminology Criteria for Adverse Events (NCI‐CTCAE, version 5.0) guides the evaluation and management of any adverse events experienced by patients.

### 2.4 Evaluations

Upon confirmation of relapse or disease progression, patients underwent comprehensive restaging, including enhanced computed tomography scans of the neck, chest, and abdomen; brain‐enhanced magnetic resonance imaging; and bone emission computed tomography. Tumor response was assessed radiologically every two treatment cycles. Two independent experienced physicians reviewed the imaging data for tumor evaluation. In cases of disagreement, a third senior physician was consulted to reach consensus. Patient safety was rigorously monitored throughout the study, with adverse events (AEs), laboratory tests, and physical examinations being recorded and evaluated during every treatment cycle.

### 2.5 Outcomes

According to RECIST 1.1 criteria, tumor response was categorized as complete response (CR), partial response (PR), stable disease (SD), or progressive disease (PD). The primary objectives of this study were to evaluate the PFS and objective response rate (ORR). PFS was defined as the time from first treatment to disease progression or death from any cause. The ORR was defined as the proportion of patients who achieved CR or PR. Disease control rate (DCR) was defined as the proportion of patients who achieved CR, PR, or SD. The secondary objectives were OS and evaluation of adverse events. OS was defined as the time from the first treatment to death or last follow‐up visit. The last follow‐up visit refers to the termination of follow‐up.

### 2.6 Statistical Analysis

Data analyses were performed using GraphPad Prism version 10.1. Categorical variables are described as frequencies and percentages. Age was compared using the Wilcoxon rank sum test. The comparison between the two groups in other variables were conducted using Fisher's exact test. OS and PFS were estimated using the Kaplan–Meier method. Statistical significance was set at *p* < 0.05.

## 3 Results

From January 1, 2023, to July 31, 2024, a retrospective analysis was conducted on 48 patients with pathologically confirmed SCLC. The patients were consecutively enrolled after meeting the inclusion criteria. The baseline demographic and clinical characteristics were in well balance between the two treatment cohorts (ANNAB and NAP) (Table 1). Baseline characteristics were generally balanced between the two groups with no statistically significant differences. However, numerical imbalances were observed in some variables, such as sensitive versus refractory disease. Given the small sample size (*n* = 48), the absence of statistical significance does not ensure full comparability between groups. Unmeasured or imbalanced prognostic factors, including but not limited to the duration of prior ICI treatment and platinum‐free interval, may have affected the efficacy outcomes. Multivariable analysis was not performed due to the limited sample size.

**TABLE 1:** Baseline characteristics of patients.

Variable	ANNAB (*n* = 24), *N* (%)	NAP (*n* = 24), *N* (%)	*p*
Age (years) median (range)	59.5 (46–75)	62.5 (45–75)	0.732
Sex			> 0.999
Male	20 (83.3)	19 (79.2)	
Female	4 (16.7)	5 (20.8)	
ECOG performance status			0.760
0	15	17	
1	9	7	
2	0	0	
Smoking history			0.815
Never	6	7	
Current	2	3	
Former	16	14	
Brain metastasis			> 0.999
No	5	6	
Yes	19	18	
Previous PCI			> 0.999
No	4	5	
Yes	18	19	
Previous radiotherapy			0.759
No	8	4	
Yes	16	20	
Brain radiotherapy			0.461
No	5	3	
Yes	19	21	
Pattern of relapse from chemotherapy			0.547
Sensitive	7	10	
Refractory/resistant	17	14	

*Note:* Age was compared using the Wilcoxon rank sum test; the other variable was compared by Fisher's exact test.

Abbreviations: ANNAB: Patients receiving nab‐paclitaxel and anlotinib. ECOG: Eastern Cooperative Oncology Group Performance Status. NAP: Patients receiving nab‐paclitaxel. PCI: Prophylactic cranial irradiation.

**FIGURE 1:**
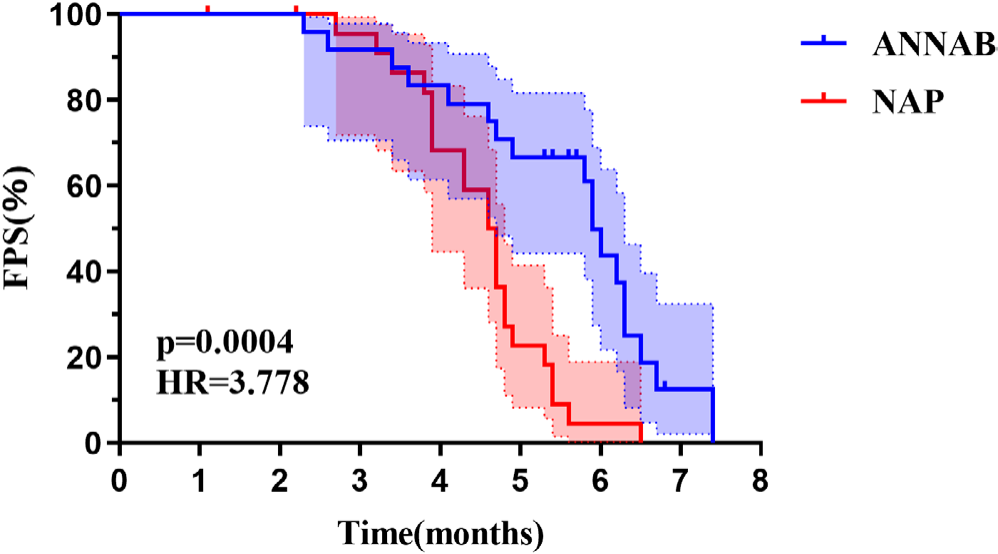
The difference of median PFS between the two groups. ANNAB: Patients receiving nab‐paclitaxel and anlotinib. NAP: Patients receiving nab‐paclitaxel. PFS: Progression‐free survival.

**FIGURE 2:**
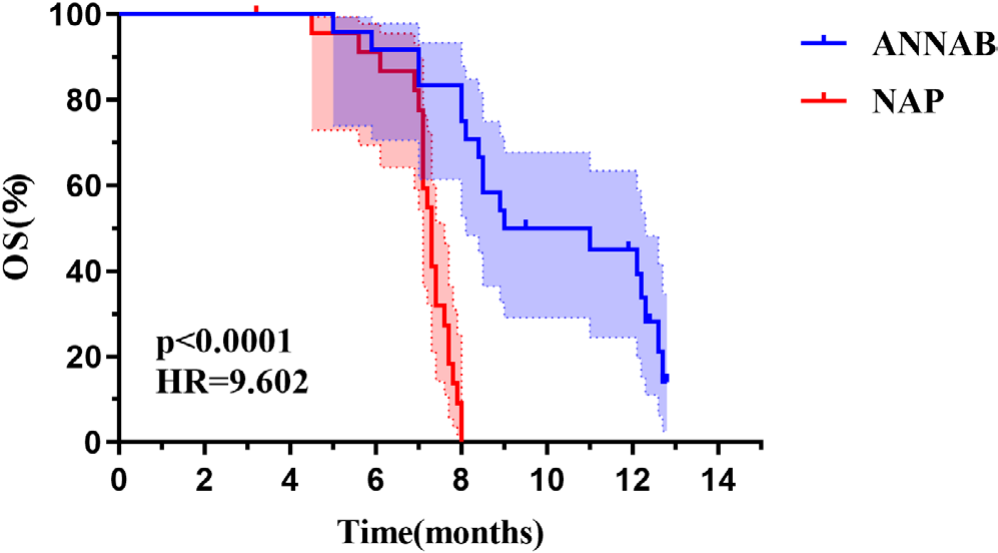
The difference of median OS between the two groups. ANNAB: Patients receiving nab‐paclitaxel and anlotinib. NAP: Patients receiving nab‐paclitaxel. OS: Overall survival.

The ANNAB treatment group demonstrated a statistically significant improvement in median PFS compared to the NAP group (6.0 vs. 4.7 months, *p* = 0.0004; Figure 1). Furthermore, the median OS was markedly prolonged in the ANNAB cohort relative to that in the NAP cohort (10.0 vs. 7.3 months, *p* < 0.0001; Figure 2).

**FIGURE 3:**
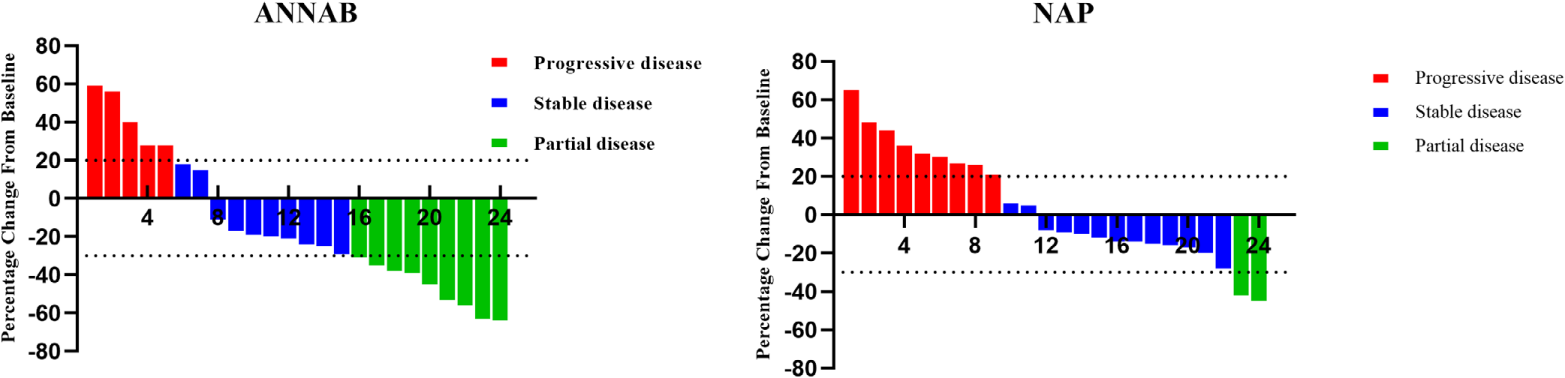
The tumor response when patients receiving different treatment schedule (according to RECIST 1.1 criteria). ANNAB: Patients receiving nab‐paclitaxel and anlotinib. NAP: Patients receiving nab‐paclitaxel.

In the ANNAB cohort, 18 patients successfully completed all six cycles of the prescribed regimen as of the last follow‐up date of January 31, 2025, eight patients in the ANNAB group remained on anlotinib monotherapy. Among the patients in the ANNAB group, nine achieved PR and 10 achieved SD, yielding a significantly higher ORR than that in the NAP group (37.5% vs. 8.3%, *p* = 0.02). A comparable DCR was observed between the two groups (79.2% vs. 62.5%, *p* = 0.34). Notably, no CR was observed in either treatment group (Figure 3).

The overall incidence of AEs was comparable between the two groups, except for peripheral neuropathy and neutropenia (Table 2). No Grade 4 AEs were observed in either group and neutropenia was the only Grade 3 AE that occurred exclusively in the ANNAB group (*n* = 1, 4.2%). The AE profile observed in this study was consistent with previously reported findings from investigations of similar treatment regimens and other therapeutic indications for these agents. In the ANNAB group, four patients required anlotinib dose reduction due to fatigue. None of the patients required a second dose adjustment. Overall, the combination regimen did not exacerbate the overall severity of adverse events compared with monotherapy.

**TABLE 2:** Comparison of treatment‐related adverse events between ANNAB and NAP groups.

Adverse event (any grade)	ANNAB (*n* = 24), *N* (%)	NAP (*n* = 24), *N* (%)	*p*
Fatigue	16 (66.7)	13 (54.2)	0.556
Anemia	9 (37.5)	4 (16.7)	0.193
Peripheral neuropathy	5 (20.8)	0 (0.0)	0.050
Hypertension	14 (58.3)	16 (66.7)	0.766
Neutropenia	7 (29.2)	1 (4.2)	0.048
Leukopenia	6 (25.0)	2 (8.3)	0.245
Vomiting	2 (8.3)	0 (0.0)	0.490
Nausea	4 (16.7)	1 (4.2)	0.348
Thrombocytopenia	3 (12.5)	1 (4.2)	0.610
Hand and foot skin reaction	16 (66.7)	10 (41.7)	0.147
Mucositis oral	7 (29.2)	5 (20.8)	0.740
Liver toxicity	3 (12.5)	1 (4.2)	0.609

*Note:* The comparison between the two groups was conducted using Fisher's exact test.

Abbreviations: ANNAB: Patients receiving nab‐paclitaxel and anlotinib. NAP: Patients receiving nab‐paclitaxel.

## 4 Discussion

This retrospective study demonstrated that the ANNAB regimen represents a potentially effective therapeutic strategy for patients with relapsed ES‐SCLC that has progressed after first‐line therapy. Compared to nab‐paclitaxel monotherapy, the ANNAB regimen significantly improved the median PFS to 6.0 months and OS to 10.0 months at the data cutoff point. These results are particularly noteworthy given the historically poor prognosis associated with relapsed SCLC, particularly in patients with primary platinum‐resistant disease, who constituted 70.8% of patients in the ANNAB group.

Although several second‐line therapeutic options are available for relapsed ES‐SCLC, their clinical benefits remain suboptimal, underscoring the need for more effective treatment strategies. Despite its category 1 recommendation for second‐line treatment of relapsed ES‐SCLC in current NCCN guidelines, the ORR of topotecan does not typically exceed 25%, and the median OS usually ranged between 6 and 9 months [14]. Lurbinectedin monotherapy also showed antitumor efficacy, which was demonstrated in a multicenter, open‐label, single‐arm Phase II trial [15]. The trial successfully achieved its prespecified primary efficacy endpoint, demonstrating a confirmed ORR of 35.2%. Unfortunately, lurbinectedin combined with doxorubicin did not demonstrate a significant improvement in OS compared with standard topotecan in the Phase III ATLANTIS trial [16]. Notably, hematological toxicity was the predominant treatment‐emergent AE of lurbinectedin, with Grade 3–4 AE including neutropenia (46%) and febrile neutropenia (5%) [15, 16]. For patients with sensitive relapse, retreatment with platinum‐based chemotherapy resulted in a significantly higher ORR (49%), especially in patients with late relapse (≥ 180 days) [17]. However, treatment‐related serious AEs such as hematologic AEs were more frequent. Amrubicin monotherapy demonstrated a significantly improved ORR compared to topotecan (31% vs. 17%, *p* < 0.001), but this enhanced antitumor activity did not translate into a statistically significant improvement in OS [18]. However, access to these agents has historically been limited in China owing to regulatory and economic barriers, posing significant challenges for their adoption as second‐line therapy. Furthermore, due to the limitations of their respective eras, the patient populations in those clinical studies had not previously received ICIs. In contrast, all patients in our retrospective cohort had prior exposure to chemoimmunotherapy. Notably, our findings demonstrate favorable ORR and PFS outcomes in this specific population.

Compared with conventional paclitaxel, nab‐paclitaxel has been shown to effectively reach the tumor microenvironment in preclinical studies, likely owing to its unique pharmacokinetic profile properties. Clinical trials have demonstrated the antitumor activity of nab‐paclitaxel in various solid tumors including lung cancer. A single‐phase 2 study from Italy investigated nab‐paclitaxel (100 mg/m [2] on Days 1, 8, and 15 every 4 weeks) as second‐line therapy for SCLC and reported modest efficacy and a tolerable safety profile. The study reported an ORR of 8% in the refractory group and 14% in the sensitive group [10]. A retrospective study from Japan evaluated nab‐paclitaxel (75–100 mg/m^2^ on Days 1, 8, and 15 every 4 weeks) as second‐line therapy and reported an ORR of 29.4% with manageable adverse events [9]. In another single‐phase 2 study evaluating the gemcitabine plus nab‐paclitaxel regimen, the combination showed an ORR of 28.1%, with a median PFS of 2.9 months and a median OS of 9.3 months [19]. Wan et al. reported that combined nab‐paclitaxel and ICIs therapy achieved a numerically higher ORR (40.7%) as second‐line therapy for ES‐SCLC. However, this regimen failed to show statistically significant improvements in PFS and OS [20]. Cumulative evidence has shown that nab‐paclitaxel‐based combination regimens seem to have superior antitumor activity compared to monotherapy as a second‐line therapy for relapsed ES‐SCLC. Notably, the aforementioned data were derived from patients who did not receive ICIs as a first‐line therapy. In contrast, our study exclusively enrolled patients treated with first‐line ICIs, thereby better reflecting the real‐world clinical practice. The combination therapy regimen demonstrated a statistically significant improvement in ORR compared to the control arm (37.5% vs. 8.3%) and significantly prolonged median PFS (6.0 vs. 4.7 months) in our study. Notably, the PFS benefit translated into a clinically meaningful OS advantage. To our knowledge, this is one of the few retrospective studies suggesting a potential OS benefit with nab‐paclitaxel plus anlotinib in patients with relapsed ES‐SCLC following first‐line chemo‐immunotherapy, although these findings require prospective validation.

The National Comprehensive Cancer Network guidelines provide limited standardized treatment options for patients with relapsed SCLC following first‐line chemoimmunotherapy. In China, anlotinib is recognized as the standard third‐line treatment for SCLC. A Phase 2 trial reported that anlotinib significantly improved PFS by 3.4 months and OS by 2.4 months compared to placebo, with an aggregate ORR of 4.9% [11]. Anlotinib is an effective second‐line therapy for patients with relapsed SCLC. Du et al. showed that anlotinib monotherapy offered an ORR of 20.0% and a median PFS of 5.6 months in a second‐line setting for relapsed SCLC [21]. Shen et al. reported the synergistic efficacy of anlotinib plus camrelizumab for ES‐SCLC as second‐line therapy, achieving an ORR of 52.9% and a median PFS of 7 months [22]. Several similar studies have demonstrated that anlotinib in combination with ICIs statistically achieved a superior ORR (varying from 37.2% to 60%) and median PFS (varying from 4.0 to 6.0 months) compared with standardized therapy.

The advent of ICIs as first‐line therapeutics has created a therapeutic lacuna in first‐line management, with current guidelines lacking consensus on standardized second‐line regimens. Our findings demonstrated that the nab‐paclitaxel–anlotinib combination regimen exhibited significantly improved PFS and ORR compared to monotherapy with either agent, suggesting a synergistic antitumor interaction. This dual‐targeted strategy—simultaneously disrupting microtubule dynamics via nab‐paclitaxel and inhibiting angiogenesis through anlotinib—represents a novel therapeutic paradigm for relapsed ES‐SCLC, addressing the critical unmet need for salvage therapies in this aggressive malignancy with limited second‐line options. Furthermore, this study enrolled patients with relapsed SCLC whose disease had progressed after initial chemoimmunotherapy.

The ANNAB regimen exhibited a favorable safety profile in this study, with only one patient developing Grade 3 hematologic toxicity. The observed incidence of nonhematologic adverse events (AEs) aligned with previously reported data from studies evaluating similar therapeutic regimens and other clinical applications of these agents. Overall, the combination regimen did not exacerbate the severity of adverse events compared with monotherapy.

Given the limited accessibility of agents such as topotecan and lurbinectedin in China, coupled with the affordability and favorable availability of anlotinib and nab‐paclitaxel in the domestic setting, this combination offers a treatment option that is both effective and tolerable, with a manageable overall safety profile.

This study has several limitations. First, this was a retrospective, nonrandomized study, and treatment allocation was based on clinical practice rather than randomization, which may lead to selection bias. Second, the single‐center design and small sample size may result in baseline numerical imbalances, and unmeasured confounding factors may affect the outcomes. Third, multivariable adjustment was not feasible due to the limited sample size.

## 5 Conclusion

This study offers novel evidence for the efficacy and safety of the ANNAB regimen as second‐line treatment for patients with relapsed SCLC after chemoimmunotherapy. Compared with the existing therapeutic options for this patient population, the ANNAB regimen demonstrated a promising efficacy profile, with a median PFS of 6.0 months and a median OS of 10.0 months. Moreover, the regimen exhibited a favorable tolerability profile with manageable adverse events. These results highlight the need for larger prospective clinical trials to validate the efficacy and safety of ANNAB regimens in this patient population.

## Author Contributions

**Li‐Jun Tian:** writing – original draft. **Fei‐Fei Zhou:** formal analysis. **Min Dai:** data curation and data analysis. **Jun‐Li Liu:** methodology. **Qi‐Sen Guo:** conceptualization.

## Funding

This work was supported by a research fund from the Shan Dong Provincial Medical Association (No. YXH2024YS030). The funding body played no role in the design, data collection, analysis, or interpretation of data.

## Ethics Statement

The study was approved by the Human Ethics Committee of Binzhou Medical University Hospital.

## Consent

All patients were eligible to participate in this study upon providing written informed consent.

## Conflicts of Interest

The authors declare no conflicts of interest.

## Data Availability

The data that support the findings of this study are available on request from the corresponding author. The data are not publicly available due to privacy or ethical restrictions.
